# Stereoisomeric Separation of Flavonoids by Two‐Dimensional Supercritical Fluid Chromatography: Identification of Adequate Chiral Columns and Application to Honey Analysis

**DOI:** 10.1002/chir.70058

**Published:** 2025-11-10

**Authors:** Laurine Réset, Bibi Ousseni, Mélodie Degrelle, Clément De Saint Jores, Caroline West

**Affiliations:** ^1^ Université d'Orléans, CNRS UM7311, ICOA Orléans France

**Keywords:** flavonoids, honey, natural products, supercritical fluid chromatography, two‐dimensional chromatography

## Abstract

Chiral flavonoids are frequently encountered in natural products consumed as food or therapeutic products. However, the taste and other bioactivities of stereoisomers are known to differ. Hence, stereoseparation methods are necessary to resolve these isomers, whether to measure stereoisomeric ratios or to purify individual stereoisomers for activity testing. In this study, we looked for the best conditions to resolve different families of chiral flavonoids, including auronols, flavanonols, aglycon, and glycosylated flavanones. Furthermore, we aimed to include this stereoseparation as the second‐dimension method in a two‐dimensional supercritical fluid chromatography system (SFC‐SFC), to achieve the resolution of chiral flavonoids in complex samples. First, a set of 10 standards of chiral flavonoids was examined on 5 immobilized polysaccharide chiral stationary phases. With the help of Derringer desirability functions, the columns offering the best options for rapid resolution were identified. Then, a sample of honey was analyzed in an SFC‐SFC system in heart‐cutting mode. The first‐dimension achiral separation resolved the complex mixture of honey and isolated the fraction of pinocembrin enantiomers, which were re‐analyzed in the second‐dimension chiral separation to measure the enantiomeric excess, and resolve co‐eluting isobaric species. This experiment demonstrates the relevance of this strategy in the resolution of flavonoid enantiomers from complex samples.

## Graphical abstract

The best enantioselective stationary phases to resolve chiral flavonoids were identified. Two‐dimensional heart‐cut supercritical fluid chromatography (SFC‐SFC) was used to isolate and resolve the enantiomers of pinocembrin from a complex sample of honey.

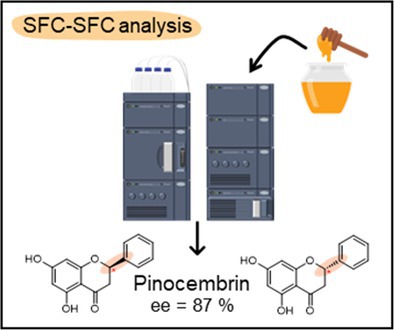

## 1 Introduction

Chirality is a significant factor in the bioactivity of chiral flavonoids [1, 2]. The latter are natural polyphenolic compounds frequently found in complex natural and food matrices such as honey [3, 4]. The stereoisomers of a chiral flavonoid may have very different tastes [5], pharmacological properties, biological interactions, or bioavailability, making their separation and individual characterization essential to improve understanding of these effects [6]. Different chromatographic methods have been proposed for this purpose, mostly in the liquid phase (LC). While derivatization followed by achiral reversed‐phase analysis was long employed, it has now been largely supplanted by direct resolution on a chiral stationary phase, where polysaccharide and cyclodextrin selectors are largely dominating the field [7]. In this context, supercritical fluid chromatography (SFC) is emerging as a promising environmentally friendly technique, combining high resolution with speed, particularly for chiral separations [7, 8, 9].

The success of an enantioselective SFC method largely depends on the judicious choice of the chiral stationary phase [10, 11]. A wide range of chiral selectors is available to interact selectively with stereoisomers, based on bonded ligands [12, 13], macrocycles [14] or polymers. Among those, the most used in chiral SFC are based on the natural polymers of cellulose or amylose derivatives, which were initially introduced by Okamoto and co‐workers [15, 16], and were firstly commercialized by Daicel Corporation and Chiral Technologies. The older phases mainly comprised non‐halogenated aromatic groups, while more recently commercialized phases developed by Chankvetadze and co‐workers [17] included chlorinated ligands to diversify the interactions between chiral analytes and chiral selectors, thereby offering alternate selectivity [18]. In addition, later developments proposed by Francotte and co‐workers [19] improved column stability through the immobilization of the polysaccharides. Indeed, older versions of these stationary phases were simply coated on the modified silica surface, rendering them prone to column bleeding in strong solvents. On the contrary, the modern, immobilized versions are much more stable, thus offering more possibilities for mobile phase optimization [20]. As a result, there is now a large diversity of such stationary phases available to increase the probability of achieving chiral resolution for any pair of stereoisomers. However useful these stationary phases may be, the predictability of a separation is still rather poor, making the screening of chiral columns an essential step in method development [21].

To rationalize the choice of a stationary phase to resolve multiple flavonoid stereoisomers in a timely fashion and with good chromatographic quality, a multi‐criteria approach based on the Derringer and Suich method is appropriate [22]. This method allows several analytical responses to be weighted according to their significance, offering a versatile strategy that is adapted to the complexity of the chromatographic parameters involved. Such desirability functions can be used for column screening [23, 24] or analysis conditions [25, 26].

Moreover, flavonoids in natural products may be included in samples of high complexity. Consequently, the simple use of one chiral column would not necessarily be sufficient to resolve the targeted stereoisomers from all other compounds present in such samples. For this reason, it is useful to include the stereoseparation method in a two‐dimensional chromatographic system, where the first dimension may be an achiral analysis, serving to isolate the target flavonoid stereoisomers, and the second dimension would serve to resolve the stereoisomers. Therefore, a heart‐cut SFC‐SFC instrument we had previously developed [27] was used in the present study to demonstrate the feasibility of this approach in the analysis of a complex honey sample.

## 2 Materials and Methods

### 2.1 Chemicals and Samples

HPLC‐grade methanol used as the mobile phase and sample diluent, was purchased from VWR (Fontenay‐sous‐Bois, France). CO_2_ with a purity of 99.7% was delivered by Air Liquide (Paris, France). Ultra‐pure water was supplied by a Milli‐Q IQ 7000 system from Merck (Darmstadt, Allemagne). Methanesulfonic acid (MSA) was purchased from Sigma–Aldrich (Saint‐Quentin‐Fallavier, France). Hydrochloric acid was purchased from ThermoFisher Scientific (Illkirch‐Graffenstaden, France).

The flavonoid standards were kindly provided by Extrasynthese (Genay, France): pinocembrin, naringenin, hesperetin, pinocembroside, naringenin‐7‐O‐glucoside, naringin, neohesperidin, taxifolin, dihydromyricetin, and alphitonin. As will appear in the following, the stereoisomeric ratio was unknown at this stage.

The honey sample was produced in the territorial collectivity of French Guiana and harvested in the commune of Remire Montjoly in December 2023. It is classified as forest honey.

### 2.2 Sample Preparation

Flavonoid standard solutions were prepared at a concentration of 1 mg/mL in MeOH.

Honey polyphenols were extracted by solid‐phase extraction (SPE) following a procedure adapted from previous works [28] as follows: 3 g of honey was dissolved in 10 mL of Milli‐Q water acidified with HCl to pH = 2. The sample was then stirred magnetically at room temperature for 10 min and filtered through a 0.45 μm filter. The SPE C18 cartridge (Starta C18‐U, 55 μm, 70 Å, 500 mg/6 mL, Phenomenex) was preconditioned with 10 mL MeOH followed by 10 mL Milli‐Q water acidified to pH = 2. The sample was then passed through the cartridge at a flow rate of 2 mL/min. The cartridge was then rinsed with 10 mL of Milli‐Q water acidified to pH = 2 to remove undesirable compounds, then 10 mL of MeOH eluted the polyphenols. The extract was then evaporated to dryness and reconcentrated in 0.5 mL MeOH for analysis.

### 2.3 Supercritical Fluid Chromatography (SFC)

#### 2.3.1 Instrumentation

The supercritical fluid chromatography system was a Waters Corporation (Milford, MA, USA) ACQUITY Ultra Performance Convergence Chromatography (UPC^2^). It was equipped with an autosampler with a partial loop volume injection system, an automated back‐pressure regulator (BPR), a column oven compatible with two 150‐mm length columns, via column switching valves. The system also included a binary solvent delivery pump compatible with a maximum mobile phase flow rate of 4 mL/min and a maximum pressure of 414 bar. Two detectors were available: a diode‐array detector (ACQUITY PDA) and a single‐quadrupole mass spectrometer equipped with an electrospray ionization source (ACQUITY QDa). An isocratic solvent manager was used as a make‐up pump and was positioned before a flow splitter, where one portion of the flow was directed to the mass detector and the other was sent to the back‐pressure regulator.

Empower 3 was used for chromatographic acquisition and for data treatment.

#### 2.3.2 Analysis Conditions

**FIGURE 1:**
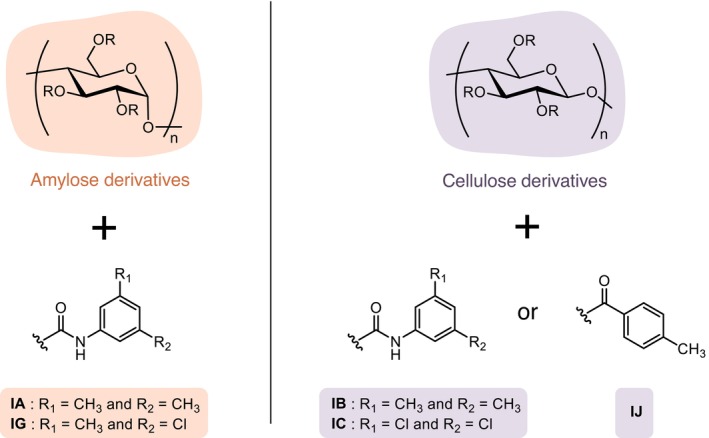
Structures of the CHIRALPAK IA‐3, IB‐3, IC‐3, IG‐3, and IJ‐3 compared in this paper.

For the analysis of all the standards, different immobilized polysaccharide stationary phases were obtained from Chiral Technologies (Illkirch‐Graffenstaden, France). CHIRALPAK IA‐3, IB‐3, IC‐3, IG‐3 and IJ‐3 (150 × 4.6 mm, 3.0 μm) were used. Their structures are presented in Figure 1. The mobile phase used was CO_2_ with MeOH. The injection volume was 2 μL. The screening conditions used for IG‐3 (gradient 1.b.) and for the other columns (gradient 1.a.) are presented in Table 1.

**TABLE 1:** Gradient profiles used at all stages for column screening or for SFC–SFC analysis of the honey sample.

Gradient 1				Gradient 2			
Time (min)	Co‐solvent (%)	Back‐pressure (bar)	Flow rate (mL/min)	Time (min)	Co‐solvent (%)	Back‐pressure (bar)	Flow rate (mL/min)
a. Gradient used for IA, IB, IC, IJ Columns				a. First‐dimension method			
0	5	150	2.0	0.0	2	150	1.7
0.5	5			1.0	2	150	1.7
12	50			7.0	30	130	1.7
13	50			10.0	90	110	0.6
13.1	5			11.0	90	110	0.6
14	5			12.0	60	130	0.9
15	5			13.5	30	150	1.2
				15	2	150	1.5
				16	2	150	1.7
b. Gradient used for IG column				b. Second‐dimension method			
0	5	150	2.0	0	5	150	2.0
0.5	5			0.5	5		
25	100			12	50		
27	100			13	50		
27.5	5			13.1	5		
28	5			14	5		
30	5			15	5		

For the analysis of the honey sample in SFC‐SFC‐DAD‐MS, two different columns were used. The first‐dimension (dim1) column was ACQUITY UPC^2^ Torus DEA (100 × 3.0 mm, 1.7 μm) from Waters to isolate the target flavonoids (pinocembrin enantiomers) from the other compounds in the honey sample. The second‐dimension (dim2) column was CHIRALPAK IG‐3 (150 × 4.6 mm, 3.0 μm) from Chiral Technologies (Illkirch‐Graffenstaden, France). This served to separate the enantiomers of pinocembrin. The mobile phase used was CO_2_ with MeOH containing 0.1% MSA for dim1, and only MeOH for dim2. The injection volume was 5 μL. The gradient programs, including variation of solvent composition, back‐pressure and flow rate, are presented in Table 1‐Gradient 2.

The column oven was heated to 30°C and the sample compartment was kept at 10°C. All chromatograms were recorded in the 210–400 nm range, with 1.2 nm resolution. Scan rate was 20 Hz. Visualization and peak integration were done at 280 nm for standards and sample.

#### 2.3.3 Mass Spectrometry Conditions

The previously optimized MS parameters were as follows [29]: make‐up fluid was MeOH, pumped at 0.4 mL/min; probe temperature 600°C; ion source temperature 120°C; cone voltage 10 V; capillary voltage +/− 0.3 kV; scan rate 3.7 Hz. The mass range for total ion recording was set from *m/z* 100 to 1000. The honey sample was analyzed in dim2 with single‐ion monitoring (SIM) mode to target pinocembrin at *m/z* 257.

## 3 Results and Discussion

### 3.1 Column Screening

#### 3.1.1 Set of Compounds

**FIGURE 2:**
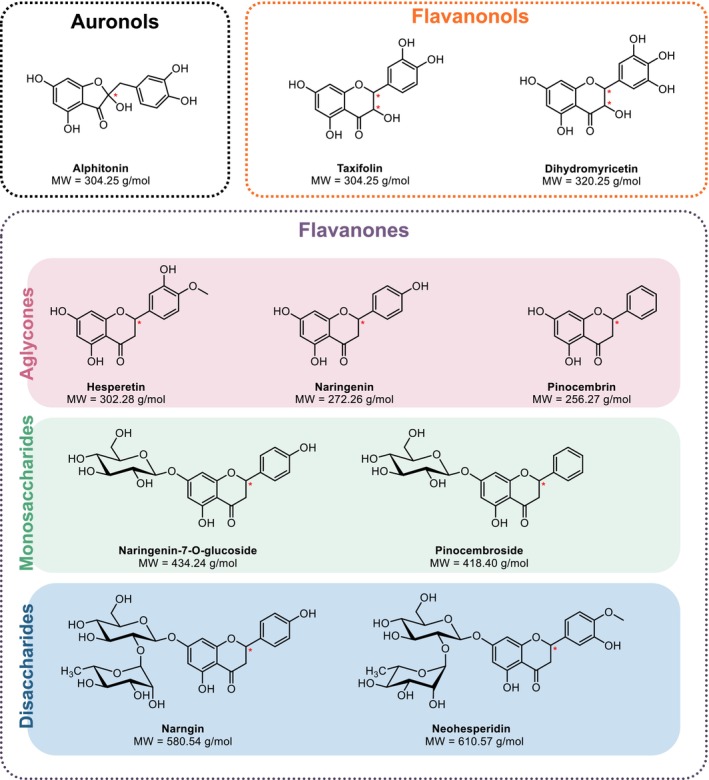
Set of flavonoid standards.

A set of 10 flavonoid compounds was selected for the column screening. This set consisted of three major classes of chiral flavonoids: auronols, flavanonols, and flavanones (Figure 2). The flavanones selected included a wide structural diversity with aglycon structures (hesperetin, naringenin, pinocembrin) and glycosylated structures comprising monosaccharide (naringenin‐7‐O‐glucoside, pinocembroside) and disaccharide moieties (naringin, neohesperidin). While the aglycon flavonoids in this selection comprise one or two chiral centers, the glycosylated ones contain many more chiral centers in the sugar moieties. For this reason, aglycon molecules would yield one or two pairs of enantiomers while the glycosylated molecules would yield a pair of diastereomers, only considering the chiral center on the genin root. Strictly speaking diastereoisomers could be resolved in an achiral environment. However, in our experience, the diastereoisomers of flavonoids are mostly unresolved on achiral columns.

#### 3.1.2 Definition of the Desirability Functions

As part of the development of a chiral separation method applied to flavonoids, a multi‐criteria approach was employed using so‐called Derringer desirability functions. The aim was to identify the columns that would perform well in separating flavonoid stereoisomers, ideally identifying one fit‐for‐all column, or possibly revealing different columns for different classes of chiral flavonoids. Derringer functions make it possible to transform several analytical responses, initially expressed in different units or different modalities, into a single standardized value enabling different chromatographic conditions to be compared. To achieve this, it is first necessary to define the comparison criteria and translate them into functions yielding corresponding d_i_ values.

**FIGURE 3:**
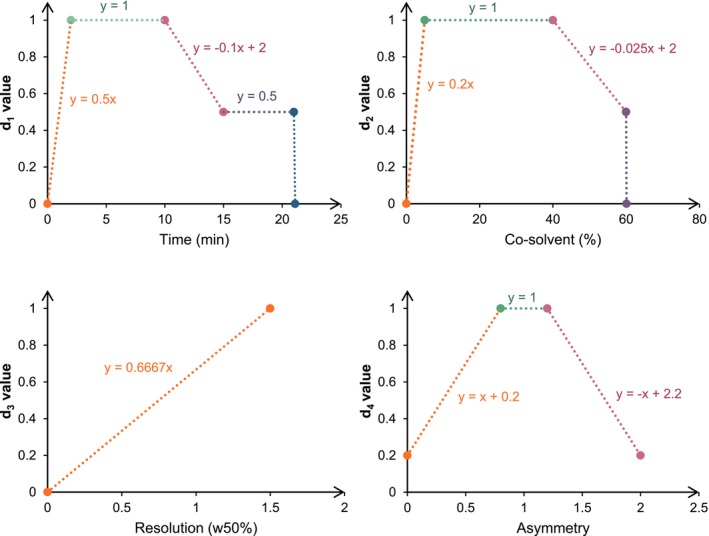
Derringer functions based on: retention time (d_1_), elution composition (d_2_), resolution calculated with width at half‐height (w50%) (d_3_) and EP asymmetry at 10% peak height (d_4_).

Four criteria were selected: retention time and proportion of co‐solvent at the moment of analyte elution for the second peak, resolution (calculated from the measured width at half‐height w50%), and peak asymmetry (measured at 10% of peak height). Each of these criteria was modeled using a desirability function, ranging from 0 (unacceptable result) to 1 (ideal result). They are presented in Figure 3 and further described below.

##### 3.1.2.1 Retention Time (d_1_)

The first criterion was retention time, represented by the d_1_ function. The retention time was considered optimal when comprised between 2 and 10 min. Below this range, the time was considered too short to guarantee effective separation, while above 10 min, a progressive penalty was applied to avoid lengthy analyses. Beyond 20 min, the d_1_ value was set to zero as the analysis was considered too long.

##### 3.1.2.2 Elution Composition (d_2_)

The second criterion was the composition of the co‐solvent at the moment of analyte elution, represented by the d_2_ function. This was also evaluated according to a three‐zone function. Values between 10% and 40% were considered ideal, as they ensure a good balance between solubility (to favor good peak shapes), elution and interaction with the chiral selector. Below 10%, desirability increased progressively, while above 40% it decreased. A limit value was set at 60%, beyond which desirability would be zero. Note that such conditions could only be reached on the IG column, because its high retentivity made it necessary to further increase the co‐solvent proportion.

In addition to analytical‐scale considerations, when seeking stereoisomer purification, this composition would also ensure good solubility to favor productivity, while retaining an acceptable volume of solvent to use, evaporate and send to waste. Low solvent proportion would be unfavorable to high productivity in preparative separations, because solubility would be impaired. On the opposite, high solvent proportion would be undesirable for economic and ecological reasons.

##### 3.1.2.3 Resolution (d_3_)

The third criterion considered was resolution, represented by the d_3_ function. The resolution between two peaks is of course the most important criterion in chiral separations. This criterion was thus heavily weighted in the overall desirability formula, as it directly determines the quality of the chiral separation. It was modeled by an increasing linear function, where the maximum d_3_ value of 1 was set at the theoretical value of baseline resolution (1.5). Above 1.5, the d_3_ value would remain equal to 1. This model reflects the idea that a gain in resolution beyond baseline resolution does not bring any additional benefit in the case of analytical‐scale separations. An optimization function for preparative‐scale separations may have adopted a different threshold, since overloading conditions usually employed at preparative scale would always result in larger peaks, thereby requiring higher resolution to ensure good peak purity.

##### 3.1.2.4 Asymmetry (d_4_)

The final criterion was asymmetry, represented by function d_4_. Asymmetry was measured at 10% of the peak height. Ideal asymmetry is expected to be in the 0.8–1.2 range, reflecting a balanced peak shape with no excessive head or tail drag. Peaks that are too asymmetric may be linked to poor analyte solubility, adsorption phenomena, column overloading, or solvent/stationary phase incompatibility. The d_4_ function was thus constructed to give maximum desirability (1) in the target range, between 0.8 and 1.2. Outside this range, the function tends towards zero. However, the minimum d_4_ value was set at 0.2 instead of 0, so as not to penalize visible but distorted peaks too much.

##### 3.1.2.5 Total Desirability (D)

These four individual desirability functions were combined to calculate an overall score, called total desirability, denoted D. It was calculated using a weighted arithmetic mean, taking into account the relative importance of each criterion, with a much heavier weighting put on resolution (factor 6), in line with the importance it has in the outcome of a chiral separation.

Thus, total desirability was calculated using Equation (1) below:

(1) $$D=\frac{d1+d2+d3\times 6+d4}{9}$$

This overall score provided an objective comparison of the chiral columns tested to separate flavonoid stereoisomers. Similarly, it could be used to compare different operating conditions to quickly identify the most promising ones, taking into account the trade‐offs between analysis speed, solvent consumption, resolution and peak quality.

#### 3.1.3 Columns Ranking Based on Flavonoids Families

**FIGURE 4:**
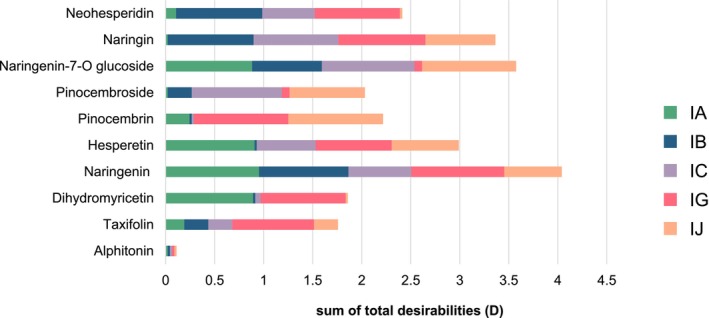
Cumulated total desirability values measured for each compound on the 5 columns tested (IA, IB, IC, IG, IJ).

**FIGURE 5:**
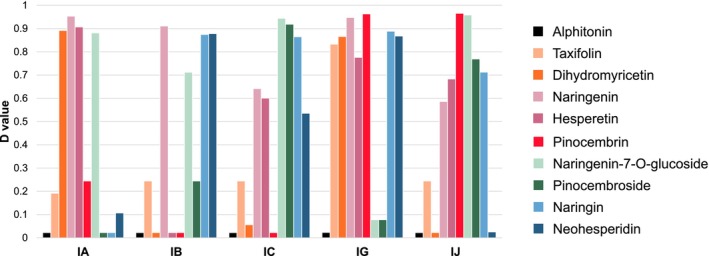
Individual total desirability values measured for each test compound on all 5 columns (IA, IB, IC, IG, IJ).

The best columns, defined by the best average performance on the four identified criteria, will then be those with the highest D values. The results are shown in Figures 4 and 5. The detailed values of the four desirability functions (d_1_, d_2_, d_3_, and d_4_) for each column and each compound, are detailed in Table S1.

First, Figure 4 shows the sum of the total desirability (D) values obtained for each flavonoid compound analyzed on the five chiral columns tested (IA, IB, IC, IG, IJ). This cumulative representation reveals the relative easiness of resolving certain flavonoids compared to others. Indeed, some flavonoids had high desirability profiles spread over several columns, indicating good overall compatibility with different chiral phases. This was the case for naringenin and its glycosylated forms. Conversely, compounds such as taxifolin, dihydromyricetin, and pinocemboside had low overall values, indicating that enantioresolution was achieved only on one or two columns. Finally, alphitonin, the only auronol in this test set, could never be resolved, raising questions about the composition of the standard, possibly comprising a single enantiomer, as it seems unlikely that none of the chiral selectors could have (even partly) resolved them.

Secondly, another way of representing these results is shown in Figure 5, which is more focused on columns, to reveal those that were most successful for certain categories of compounds.

The IG column was the best performing overall, with high values of overall desirability for the majority of compounds. It was particularly effective for non‐glycosylated flavonoids, both flavanones and flavanonols, with an overall mean desirability of 0.8. It was also suitable for flavanones with a disaccharide moiety (blue). However, and somewhat surprisingly, it was unsuccessful for flavanones glycosylated with monosaccharide groups (green), with less than 0.1 total desirability. The IG column therefore has a more favorable chiral interaction capacity for the flavonoid structures tested.

The IJ column showed an intermediate profile. It was effective for most of the flavanone family but not for flavanonols. It worked well for aglycone forms (purple and red) and flavanones with a monosaccharide group (green). However, it was less successful for flavanones with a disaccharide group (blue) as it resolved the diastereoisomers of naringin but not those of neohesperidin.

The IC column performed well for glycosylated flavanones (green and blue) with a total *D* value of around 0.9 for monosaccharides and 0.7 for disaccharides. But it remained limited for the other compounds.

Conversely, the IA and IB columns showed more erratic behavior. The IB column performed particularly well for flavanones with a disaccharide group (*D* = 0.88) (blue) and moderately well for compounds with a monosaccharide (*D* = 0.47) (green). However, it is ineffective for other compounds. The IA column was suitable for the aglycone forms of flavanones and flavanonols, but not for glycosylated structures, except for naringenin‐7‐O‐glucoside.

Interestingly, reverse effects were observed between certain columns. For example, compounds resolved on the IA column were not well resolved on the IB column, and conversely, even though these two columns have the ligands but are based on different polymers (Figure 1). A similar observation can be made for the IC/IG column pair, which both carry chlorinated ligands, although slightly different. As was previously observed in many occasions, this suggests that the nature of the amylose or cellulose polymer constituting the stationary phase strongly influences the possibility to resolve stereoisomers. In this study, the amylose‐based phases appeared generally more selective for aglycone forms, while those based on cellulose showed better selectivity for glycosylated forms.

Overall, this analysis highlights the benefits of using different columns within the same compound family. The total desirability approach is an objective tool for guiding the choice of columns, particularly during the analytical optimization phase or the development of robust chiral methods.

### 3.2 Honey Sample Application

Having identified the best columns in relation to flavonoid structure, we proceeded to examine possible applications. Complex natural samples were of particular interest as they contain many different species, and usually include many isomeric compounds. In such cases, even when flavonoids could be partly isolated through selective extraction or sample preparation procedures, they would still contain many compounds with high structural similarity. It is therefore unlikely that a single chiral column would be able to resolve the stereoisomers of a particular flavonoid from all other species. As a result, a two‐dimensional strategy was considered, where the first dimension would comprise an achiral stationary phase to start resolving all species, isolate target flavonoids from other species, then the second dimension would comprise the chiral column to resolve the stereoisomers.

To demonstrate this idea, a sample of honey from French Guiana was analyzed. The sample was expected to contain a variety of flavonoids, including pinocembrin, an aglycone flavanone derived from propolis (Figure 2), which was often described in the phenolic composition of honey [30, 31, 32, 33]. As appears in Figure 5, pinocembrin, shown in red, was well resolved on IG and IJ columns, where the total desirability values were close to 1. Although the IJ column was relevant, the IG column was selected for its broader applicability on different sorts of flavonoids. Full results of retention times and resolution measured on this column are provided in Table S2. In the literature, pinocembrin enantiomers had been previously resolved in reversed‐phase liquid chromatography (LC) on Chiralpak AD‐H [34], which is the coated version of the IA column examined in the present paper, but also in normal‐phase LC on Chiralpak AS‐H and on Chiralcel OD‐H, which is the coated equivalent of the IB column [35]. Bee pollen flavonoids were previously resolved in SFC‐UV on the Chiralpak AD‐H column, but pinocembrin was not identified in this study [8].

The two‐dimensional SFC‐SFC system with diode‐array and mass spectrometric detection had been previously developed [27]. The first‐dimension method was based on a previously developed method for flavonoid analysis [29]. The gradient programs are described in Table 1 (Gradient 2).

**FIGURE 6:**
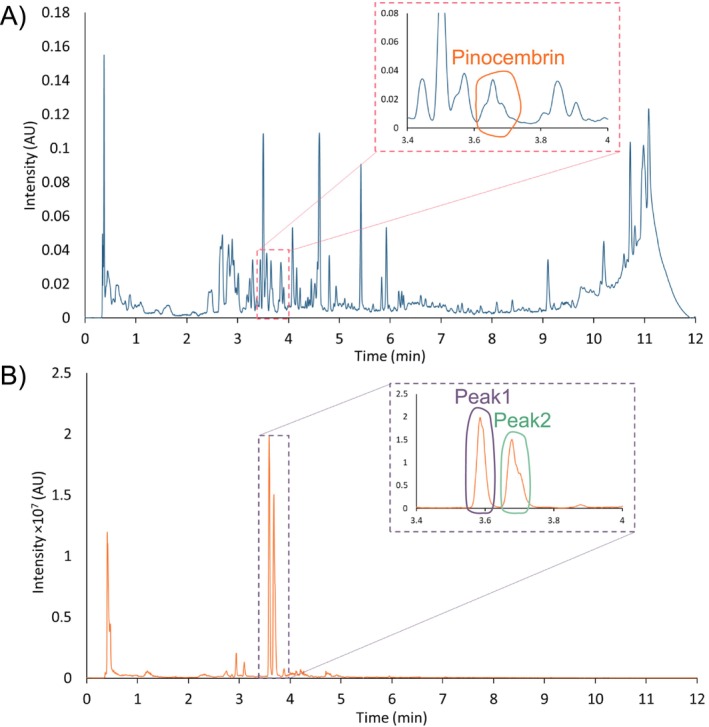
1D chromatograms of honey sample. (A) SFC‐UV analysis (280 nm). (B) SFC‐MS analysis (*m/z* 257). Analysis conditions (dim1): ACQUITY UPC^2^ Torus DEA (100 × 3.0 mm, 1.7 μm), co‐solvent: MeOH + 0.1% MSA, 30°C, 150–110 bar, 1.7–0.6 mL/min (gradient conditions in Table 1, gradient 2.a).

Although SPE was applied to extract polyphenols, the honey sample was still highly complex as appears with its rich chromatographic profile in Figure 6A. It would therefore be unrealistic to determine the enantiomeric excess of flavonoids in a single dimension of separation. To determine the presence of pinocembrin, a standard was first analyzed in the first and second dimensions (dim1 and dim2) to determine the retention times in each method. As a result, the pinocembrin peak was detected in dim1 in UV at 280 nm on the diethylaminopropyl‐bonded column (Figure 6A). However, this peak was clearly not well resolved from other species. To simplify reading the chromatogram, the SIM chromatogram recorded in positive ionization mode, tracking the [M + H]^+^ ion of pinocembrin (*m/z* 257) is represented in Figure 6B. On this chromatogram, close to the retention time corresponding to pinocembrin, two peaks were visible. In order to ascertain which of them corresponded to pinocembrin, these two peaks were both collected in storage loops and re‐injected in the second‐dimension column.

**FIGURE 7:**
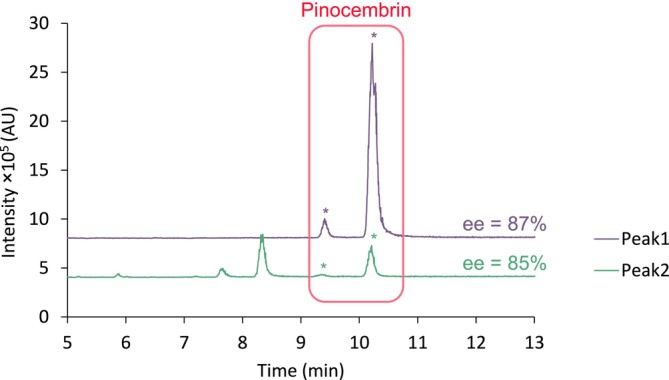
SFC‐MS (*m/z* 257) chromatograms of honey sample in dim2 corresponding to the peak of pinocembrin extracted from dim1. Analysis conditions (dim2): CHIRALPAK IG‐3 (150 × 4.6 mm, 3.0 μm), co‐solvent: MeOH, 30°C, 150 bar, 2.0 mL/min (gradient conditions in Table 1, gradient 2.b).

Injection of peak 1 in dim2 on the IG column enabled the pinocembrin enantiomers to be separated as the retention times corresponded to those of the pinocembrin standard (Figure 7). An enantiomeric excess of 87% was measured. When peak 2 was transferred from dim1 to dim2, four peaks were visible. Two of them corresponded to the two enantiomers of pinocembrin, but in smaller quantities than in peak 1. This small presence nevertheless enabled the enantiomeric excess to be calculated at 85%. This suggests that the presence of pinocembrin in peak 2 probably corresponded to a remnant of peak 1, due to their proximity in the dim1 chromatogram. In addition, a second pair of peaks was detected in the dim2 chromatogram of peak 2. As the *m/z* value was identical, these peaks were probably originating from pinocembrin isomers. For instance, dihydrodaidzein (7,4′‐dihydroxyisoflavanone) was previously described in honey [36]. Since it is an isoflavanone (B‐ring in the C3 position) while pinocembrin is a flavanone (B‐ring in the C2 position), it is reasonable to think that they could have been resolved both in the achiral column and in the chiral column. However, more confident identification would have required the analysis of a standard compound, or purification of the peaks to carry out NMR experiments, which was beyond the scope of the present study.

Finally, it is important to mention that scalemic mixtures analyzed in an achiral chromatographic system may cause self‐disproportionation of enantiomers (SDE), as was first demonstrated by Cundy and Crooks [37]. To put it simply, intermolecular associations of the enantiomers in solution may yield enantiomerically enriched or enantiomerically depleted fractions in achiral chromatography. In other words, the enantiomeric excess may vary when the front, apex and tail of the peak are examined. While this does not appear to be significant in the present example (the ee values were measured as 85% and 87% in the two fractions), it further supports the interest of collecting the full peak in a single fraction to be transferred from the first dimension to the second, in order to obtain an accurate measurement of ee. This phenomenon should also be considered to implement comprehensive methods of two‐dimensional chromatography involving an achiral step and a chiral step, where the peak containing both enantiomers eluting from the achiral column would be divided into several fractions. Further information on SDE in achiral chromatography can be found in the literature [38, 39].

## 4 Conclusion

In the above study, it was shown that certain polysaccharide chiral selectors appeared to be specific to particular flavonoid structures, including a marked difference between the amylose phases performing better for aglycone forms and the cellulose phases for glycosylated flavanones. Based on these observations, suitable columns were defined for different classes of flavonoids, using Derringer desirability functions. This made it possible to map the best‐performing column/compound combinations for separating stereoisomers.

A complex sample of French Guiana honey containing a variety of flavonoids was analyzed with heart‐cutting two‐dimensional supercritical fluid chromatography (SFC‐SFC‐DAD‐MS). The first‐dimension method was used to isolate the enantiomers of pinocembrin, a bioactive aglycon flavanone naturally present in bee products; then the second‐dimension method resolved the enantiomers, and further separated them from other isobaric compounds. Determining the enantiomeric excess provides a better understanding of the composition of the sample that would be helpful in bioactivity studies, or when biosynthetic pathways are examined.

Furthermore, these results illustrate the usefulness of a two‐dimensional chromatographic system to characterize chiral molecules in complex natural matrices. This methodology opens the way to a better understanding of the chiral profile of flavonoids present in natural products such as honey, illustrating the full potential of SFC‐SFC in advanced chiral analysis.

## Supporting information


**Table S1:** Detailed values obtained from desirability functions for each standard compound: retention time (d1), composition of the mobile phase at the moment of elution (d2), resolution calculated with width at half‐height (w50%) (d3), and asymmetry at 10% peak height (d4).
**Table S2:** Detailed values of retention times (Tr), asymmetry, elution composition (corresponding to the proportion of co‐solvent at the moment of analyte elution) and resolution observed on the IG column.

## Data Availability

The data that support the findings of this study are available from the corresponding author upon reasonable request.
