# Supplementary material for: Stereoisomeric Separation of Flavonoids by Two‐Dimensional Supercritical Fluid Chromatography: Identification of Adequate Chiral Columns and Application to Honey Analysis
Source: Chirality. 2025 Nov 10;37(11):e70058. doi: 10.1002/chir.70058 (PMC12616603; doi:10.1002/chir.70058)
Supplement: Supplementary file 1 — Table S1: Detailed values obtained from desirability functions for each standard compound: retention time (d1), composition of the mobile phase at the moment of elution (d2), resolution calculated with width at half‐height (w50%) (d3), and asymmetry at 10% peak height (d4). Table S2: Detailed values of retention times (Tr), asymmetry, elution composition (corresponding to the proportion of co‐solvent at the moment of analyte elution) and resolution observed on the IG column. [file CHIR-37-e70058-s001.pdf]

Supporting Information to:

Stereoisomeric separation of flavonoids by SFC-SFC: screening of chiral columns and application to honey analysis

Laurine Réset, Bibi Ousseni, Mélodie Degrelle, Clément De Saint Jores, Caroline West*

Université d'Orléans, CNRS, ICOA, UMR 7311, Orléans, France

Table S1 – Detailed values obtained from desirability functions for each standard compound: retention time (d_1), composition of the mobile phase at the moment of elution (d_2), resolution calculated with width at half-height (w50%) (d_3), and asymmetry at 10% peak height (d_4).

		Auronols	Flavanonols		Flavanones						
		Alphitonin	Taxifolin	Dihydromyricetin	Naringenin	Hesperetin	Pinocembrin	Pinocembroside	Naringenin-7-O glucoside	Naringine	Neohesperidin
**IA**	d1	0.50	0.82	0.97	1.00	0.76	1.00	0.00	0.88	0.50	0.44
	d2	0.50	0.78	0.92	1.00	0.91	1.00	0.00	0.88	0.48	0.74
	d3	0.00	0.00	1.00	1.00	1.00	0.00	0.00	1.00	0.00	0.00
	d4	0.20	0.20	0.20	0.58	0.54	0.20	0.20	0.42	0.20	0.32
	D	0.02	0.19	0.89	0.95	0.91	0.24	0.02	0.88	0.02	0.11
**IB**	d1	0.50	0.50	0.47	1.00	0.50	0.50	1.00	1.00	0.90	0.90
	d2	0.50	0.50	0.44	1.00	0.50	0.50	1.00	1.00	0.84	0.84
	d3	0.00	0.00	0.00	1.00	0.00	0.00	0.00	0.70	1.00	1.00
	d4	0.20	0.20	0.20	0.20	0.20	0.20	0.20	0.20	0.20	0.20
	D	0.02	0.24	0.02	0.91	0.02	0.02	0.24	0.71	0.87	0.88
**IC**	d1	0.50	1.00	0.50	1.00	1.00	0.50	1.00	0.97	0.79	0.68
	d2	0.50	1.00	0.50	1.00	1.00	0.50	0.96	0.92	0.73	0.63
	d3	0.00	0.00	0.00	0.60	0.53	0.00	1.00	1.00	1.00	0.56
	d4	0.20	0.20	0.50	0.20	0.20	0.20	0.33	0.66	0.34	0.20
	D	0.02	0.24	0.06	0.64	0.60	0.02	0.92	0.94	0.86	0.54
**IG**	d1	0.50	0.53	0.79	0.88	0.27	0.99	0.50	0.50	0.96	0.80
	d2	0.50	0.26	0.75	0.83	0.25	0.97	0.00	0.00	0.91	0.75
	d3	0.00	1.00	1.00	1.00	1.00	1.00	0.00	0.00	1.00	0.98
	d4	0.21	1.00	0.48	0.88	0.99	0.75	0.20	0.20	0.20	0.51
	D	0.02	0.83	0.87	0.95	0.78	0.96	0.08	0.08	0.89	0.87
**IJ**	d1	0.50	0.50	0.00	1.00	1.00	1.00	1.00	1.00	1.00	0.50
	d2	0.50	0.50	0.00	1.00	1.00	1.00	1.00	1.00	1.00	0.50
	d3	0.00	0.00	0.00	0.51	0.66	1.00	0.73	1.00	0.70	0.00
	d4	0.20	0.20	0.20	0.20	0.20	0.69	0.53	0.63	0.20	0.23
	D	0.02	0.24	0.02	0.59	0.68	0.97	0.77	0.96	0.71	0.03

Table S2 – Detailed values of retention times (Tr), asymmetry, elution composition (corresponding to the proportion of co-solvent at the moment of analyte elution) and resolution observed on the IG column.

		IG			
Name		Tr(min)	Asym	elution composition	R°(w50%)
Naringenin	1	10.91	1.35	45.52	3.32
	2	11.51	1.29	47.85	
Hesperetin	1	14.63	1.22	59.93	17.47
	2	20.61	1.19	83.15	
Pinocembrin	1	9.36	1.44	39.51	4.80
	2	10.18	1.46	42.69	
(+/-) Taxifolin	1	14.42	0.81	59.14	2.81
	2	15.36	1.06	62.76	
Dihydromyricetin	1	10.96	2.61	45.70	8.28
	2	13.23	1.44	54.52	
Alphitonin	1	6.47	1.99	28.30	-
	2			0.00	
(+) Taxifolin	1	15.36	1.08	62.77	-
	2			0.00	
Pinocembroside	1	16.12		65.73	0.00
	2	16.46		67.04	
Naringenin-7-O glucoside	1	15.68		64.00	0.00
	2	16.03		65.37	
Naringine	1	10.09		42.35	1.89
	2	10.77		44.99	
Neohesperidine	1	11.37	1.54	47.32	*1.47*
	2	12.67	1.84	52.35	
